# Practice toward standardized performance testing of computer-aided detection algorithms for pulmonary nodule

**DOI:** 10.3389/fpubh.2022.1071673

**Published:** 2022-12-07

**Authors:** Hao Wang, Na Tang, Chao Zhang, Ye Hao, Xiangfeng Meng, Jiage Li

**Affiliations:** ^1^Division of Active Medical Device and Medical Optics, Institute for Medical Device Control, National Institutes for Food and Drug Control, Beijing, China; ^2^School of Bioengineering, Chongqing University, Chongqing, China

**Keywords:** computer-aided detection (CAD), algorithm testing, pulmonary nodule, test set, data curation

## Abstract

This study aimed at implementing practice to build a standardized protocol to test the performance of computer-aided detection (CAD) algorithms for pulmonary nodules. A test dataset was established according to a standardized procedure, including data collection, curation and annotation. Six types of pulmonary nodules were manually annotated as reference standard. Three specific rules to match algorithm output with reference standard were applied and compared. These rules included: (1) “center hit” [whether the center of algorithm highlighted region of interest (ROI) hit the ROI of reference standard]; (2) “center distance” (whether the distance between algorithm highlighted ROI center and reference standard center was below a certain threshold); (3) “area overlap” (whether the overlap between algorithm highlighted ROI and reference standard was above a certain threshold). Performance metrics were calculated and the results were compared among ten algorithms under test (AUTs). The test set currently consisted of CT sequences from 593 patients. Under “center hit” rule, the average recall rate, average precision, and average F_1_ score of ten algorithms under test were 54.68, 38.19, and 42.39%, respectively. Correspondingly, the results under “center distance” rule were 55.43, 38.69, and 42.96%, and the results under “area overlap” rule were 40.35, 27.75, and 31.13%. Among the six types of pulmonary nodules, the AUTs showed the highest miss rate for pure ground-glass nodules, with an average of 59.32%, followed by pleural nodules and solid nodules, with an average of 49.80 and 42.21%, respectively. The algorithm testing results changed along with specific matching methods adopted in the testing process. The AUTs showed uneven performance on different types of pulmonary nodules. This centralized testing protocol supports the comparison between algorithms with similar intended use, and helps evaluate algorithm performance.

## Introduction

Lung cancer has become the most common malignant tumor that threatens human health (1). Pulmonary nodules are common imaging signs in the early stage of lung cancer. Early detection of pulmonary nodules and timely medical intervention can improve the survival rate of patients (2), CT screening provides an effective method for early diagnosis, thereby accumulating tremendous amount of CT images for radiologists to read (3).

CAD algorithm for pulmonary nodules may help assist clinical decisions, and improve clinical work efficiency (4). Usually, the common function of a CAD system is to detect the location of the lesions (5). Afterwards, lesion type classification and lesion size measurement may also be covered. Therefore, lesion detection is fundamental and important. Many researchers have devoted to improving or innovating Artificial Intelligence (AI) -enabled algorithms for pulmonary nodule detection to improve the detection performance of the algorithms (6–21). Public datasets such as LIDC-IDRI (22), LNDb (23), ANODE09 (24) supported algorithm competitions and researches (25–37), which provided insights on how to arrange algorithm testing. However, the procedure of algorithm competitions is significantly different from product verification and validation, which should provide high quality evidence for regulation. In algorithm competitions, the training data and testing data may come from the same dataset (22) and have similar features. The annotation process may be conducted by clinicians from a limited number of hospitals, which may not reflect wide consensus of medical community. This situation may decrease the comparability of algorithm testing results among different labs. More effort is thus needed to improve algorithm testing procedure. And for relative research, details of the matching process between reference standard and algorithm predicted ROIs were not sufficiently described in the literature, but different matching methods may lead to differences in performance test results which limit the comparability among different algorithms.

AI-enabled CAD products for pulmonary nodules have been developed and marketed in many countries (38–40), and most of them are in the form of software as a medical device (SaMD). While stakeholders are interested to compare products and understand their quality, the verification and validation of such products is often conducted by manufacturers individually. Currently, there are differences in the performance metrics and verification methods claimed by different manufacturers, resulting in a lack of comparability between algorithms (41). There is also a lack of understanding of the common quality characteristics of these algorithms. Recently, standardization organizations start to establish the framework to conduct algorithm performance testing. It would be necessary to gain practical experience (42, 43).

In this paper, a protocol is proposed to build datasets for algorithm testing from the perspective of third party and regulation. Three matching methods [center distance (44), center hit (45), area overlap (46)] are applied to test the detection performance of AUTs provided by ten different developers. The overall performance of ten AUTs and the differences in algorithm performance under different matching methods are analyzed. Algorithm errors are also analyzed to further understand the quality features of the AUTs. This work is aimed at establishing prototypes for standardized verification of such products and promote quality control. It may provide experience for development of technical standards on CAD products.

## Materials and methods

### Test set

**Figure 1:**
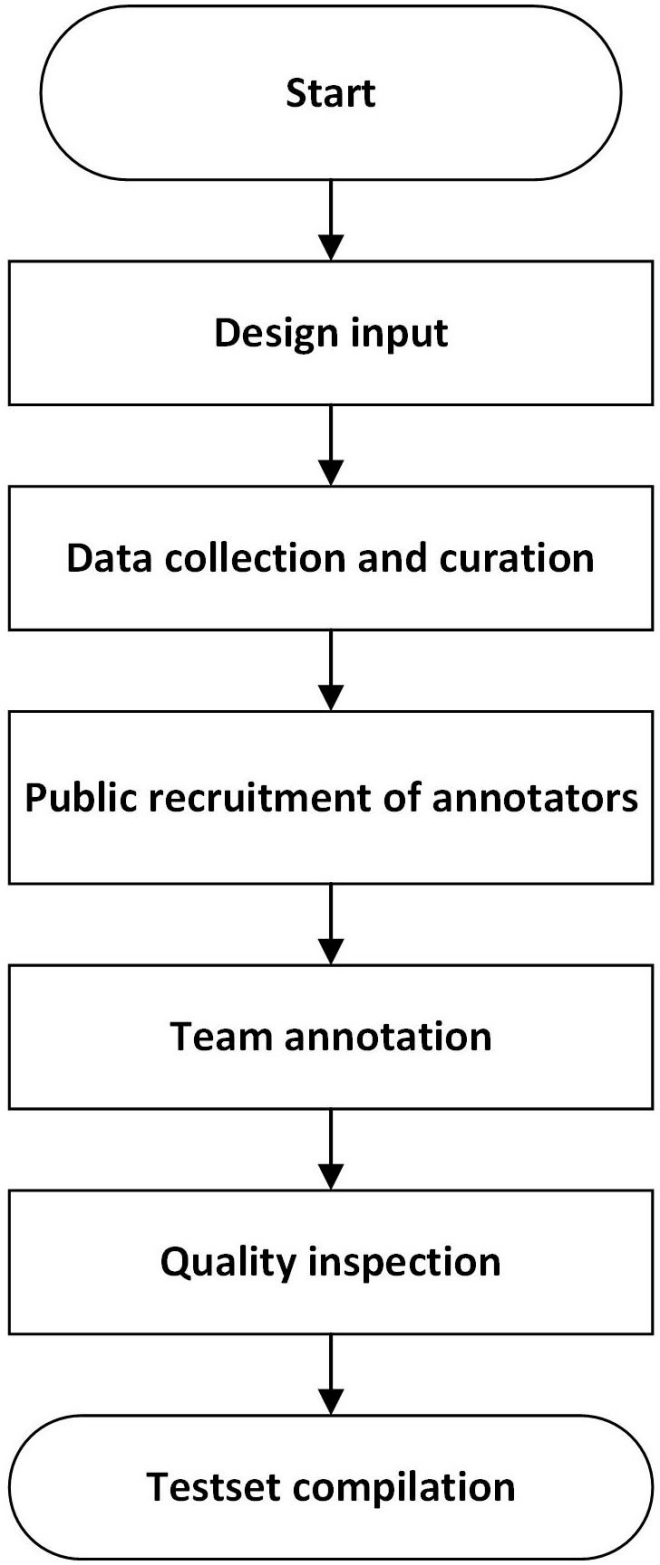
The workflow to build a test set for pulmonary nodules.

The construction of a test set followed the procedure in Figure 1, which referred to the strategy in literature (47).

The design input clarified data collection requirement (CT equipment, imaging parameters such as tube voltage, slice thickness, slice spacing and reconstruction algorithm), data diversity (patient spectrum, geological distribution of data collection sites, proportion of different types of nodules) and rules for unique data identification. More details can refer to literature (47).

CT images of patients with pulmonary nodules were collected retrospectively under local ethical approval and patient privacy protection requirements. They are stored as Dicom (Digital Imaging and Communications in Medicine) files. A data cleaning procedure was conducted to ensure the integrity and validity of CT images. Cases with discontinuous imaging sequences, missing slices, unreadable files, problematic field of view and irrelevant imaging position were excluded. Further examination was conducted to remove data which were duplicated internally or externally and ensure the uniqueness of each image sequence.

Annotation was conducted by groups of radiologists on a custom-built annotation software. CT sequences can be displayed at different angles. Multiple window level and width settings are provided. Annotators were asked to label the location (center of the bounding box), boundary and type of pulmonary nodules on each slice (usually cross-sectional view). The size of the nodule was also recorded, containing long diameter, short diameter, average diameter, length and width of the bounding box. The outputs were exported as csv files, which were used as reference standard during algorithm testing.

Annotators were publicly recruited through a qualification exam, which evaluated annotators' skills to detect and segment pulmonary nodules on 20 thoracic CT sequences in comparison with annotation results from a high-level expert panel. One hundred and eighty-five candidates from 112 hospitals participated in the test. The passing criteria is: precision >0.8, recall >0.8 and Dice coefficient >0.8. 24 junior radiologists and 15 senior radiologists passed the exam and received training. They were from 25 hospitals in 13 provinces in China. The junior radiologists have been engaged in image reading service in tertiary hospitals for more than 5 years and have a title of resident doctor or above. Every three junior radiologists form a team, and the team leader is a deputy chief physician with more than 10 years of work experience. The senior experts are chief physicians or deputy chief physicians with more than 15 years of work experience. They provide final review and arbitration.

The annotation consists of three steps. First, every team of junior radiologists independently highlight pulmonary nodules on a batch of CT image sequences back-to-back, and then a computer program automatically evaluates the consistency of the detection results. If they are consistent, the output will be the union of the results marked by the three junior radiologists. If results are inconsistent on certain slices, such slices will be highlighted to remind senior experts. Second, the same team gives the classification labels for the detected nodules from the previous step. Third, the outputs are reviewed by the leader from another team and arbitrated by arbitration experts. In case there are controversial results, the arbitration experts will discuss and determine the final annotation result.

The dataset contains a total of 593 cases from 22 hospitals in 9 provinces in China, with a total of 6,109 nodules. These nodules were counted from the perspective of type and size. The proportion of nodules of different types and sizes was shown in Tables 1, 2. A range of CT scanner manufacturers and models was represented (38% of scans from seven different Siemens Definition, Sensation, and Emotion scanner models, 36% of scans from three different Philips Brilliance, iCT scanner models, 12% of scans from three different GE Medical Systems LightSpeed, BrightSpeed scanner models, 9% of scans from UIH uCT scanners, 3% of scans from Toshiba Aquilion scanners, 2% of scans from other scanner models). Tube voltage ranged from 100 to 150 kV (mean: 117.4 kV). Tube current ranged from 17 to 544 mA (mean:189.8 mA). Conventional and enhanced CT accounted for 67% and low-dose screening CT accounted for 33%. The in-plane pixel size ranged from 0.5 to 0.9 mm. Slice thicknesses included 0.625 mm (1.2%), 0.75 mm (4.7%), 0.8 mm (14.0%), 1 mm (34.1%), 1.25 mm (13.8%), 1.5 mm (1.3%), 2 mm (28.5%), 2.5–6 mm (2.9%). 72.1% of CT scans were reconstructed using standard algorithm and lung algorithm, and 27.9% were reconstructed using high frequency algorithm and bone algorithm.

**Table 1:** The proportion of different types of nodules.

**Types**	**Proportion (%)**
Solid nodules	42.66
Part-solid nodules	5.06
Pure ground-glass nodules	19.58
Calcified nodules	7.40
Pleural nodules	23.06
Pleural calcified nodules	2.24

**Table 2:** The proportion of different sizes of nodules.

**Sizes (diameter/mm)**	**Proportion (%)**
< 4	69.91
[4, 6)	19.63
[6, 10)	7.28
≥10	3.18

The definition of various types of nodules is as follows (48):

(1) Solid nodules: focal increased density shadows with clear borders in the lung parenchyma with a circular or quasi-circular (sphere or sphere-like) boundary, and the bronchi and blood vessel edges in the lesions cannot be identified. The maximum long diameter of the nodule is ≤ 3 cm.
(2) Part-solid nodules (mixed ground-glass density nodules): focal increased density shadows with clear borders in the lung parenchyma, round or round-like (sphere or sphere-like). In some lesions, the bronchi and blood vessel edges can be identified, and the maximum long diameter is ≤ 3 cm.
(3) Pure ground-glass nodules: focal increased density shadows with clear borders in the lung parenchyma with a circular or quasi-circular (sphere or sphere-like) boundary, and the edges of the bronchi and blood vessels in the entire lesion can be identified, with a maximum long diameter of ≤ 3 cm.
(4) Calcified nodules: circular or quasi-circular (sphere or sphere-like) complete calcium deposits in the lung parenchyma with clear boundaries, the maximum long diameter is ≤ 3 cm, and the CT value is usually above 100 HU.
(5) Pleural nodules and pleural plaques: Pleural nodules are round and round-like (sphere and sphere-like) or irregular focal increased density shadows originating from the pleura, often connected to the broad base of the pleura, the maximum long diameter ≤ 3 cm. Pleural plaques are irregular flat protrusions of the pleura that are localized and broad-based, with an irregular surface. Hereinafter referred to as pleural nodules.
(6) Pleural calcified nodules: round or round-like (sphere or sphere-like) complete calcium deposition foci with clear borders originating from the pleura, the maximum long diameter is ≤ 3 cm, and the CT value is usually above 100 HU.

More details and examples of pulmonary nodule annotation refer to an expert consensus (48).

The dataset is managed as a sequestered test set according to the IEEE 2801–2022 standard (49).

### Algorithm under test (AUT)

10 AUTs provided by 10 different developers are tested in this study. The developers are from medical device industry. They all use deep convolutional neural network to perform lung nodule detection. The AUTs are generally developed by transfer learning based on developers' self-developed training sets. The pre-training models referred to Faster-RCNN, YOLO and other public available algorithms (50–52). 9 AUTs are designed to detect 2-D targets on each CT image. 1 AUT is designed to detect 3D targets. To protect confidential information, details on the algorithm architecture and weights are not disclosed. According to the intended use, 3 AUTs detect only pulmonary nodules (4 types in total), and 7 AUTs detect 4 types of pulmonary nodules and 2 types of pleural nodules (6 types in total). The output of the AUTs highlight the region of predicted nodule in the form of a bounding box (B-Box) on each slice.

During the test, each developer provided a server to install and run their AUT. The configuration of the server was determined by the developer. No internet connection was allowed. The test set was imported to the AUT through an external hard drive.

### Mark-labeling methods

**Figure 2:**
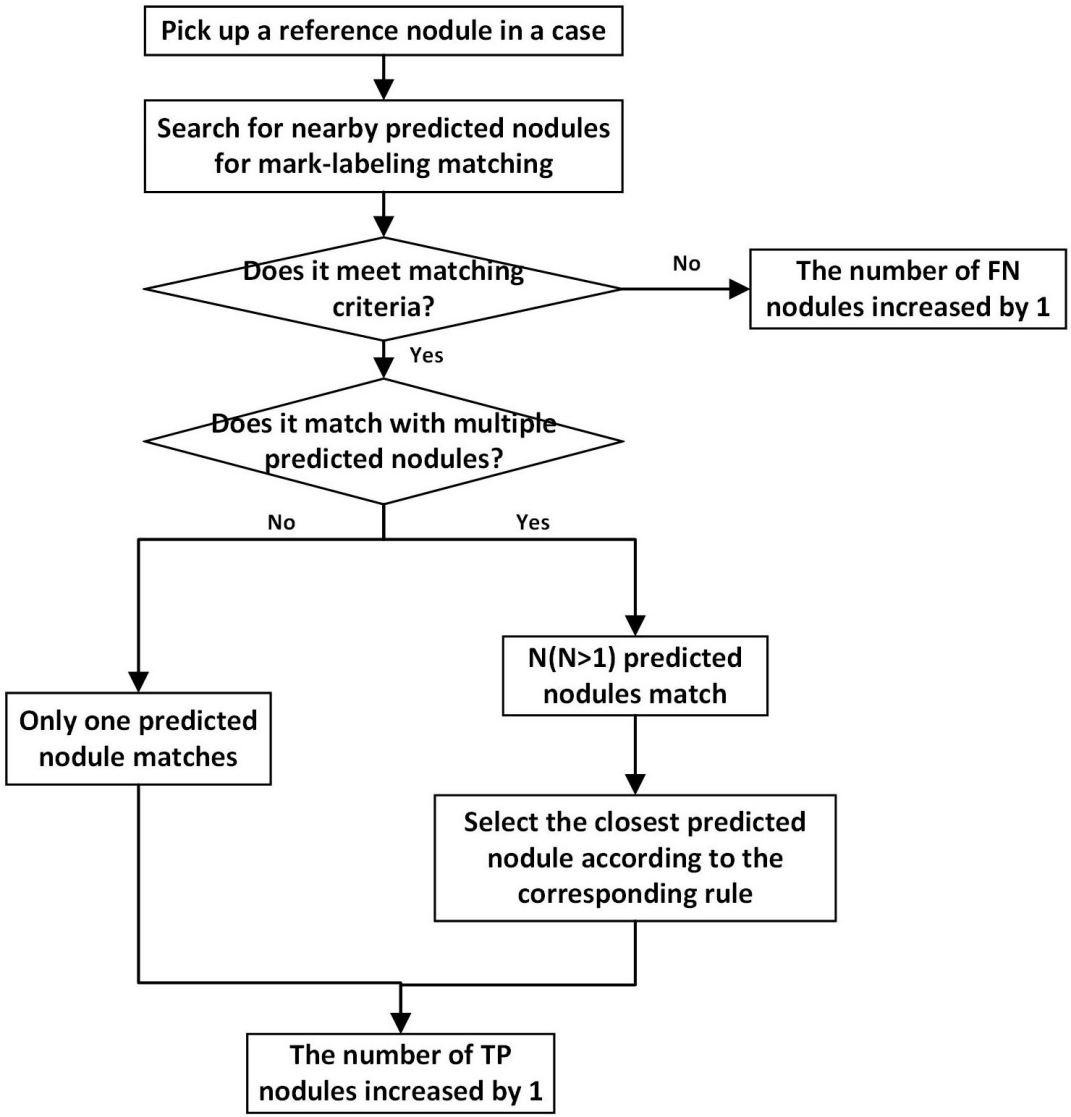
The flowchart depicting matching process for one reference nodule. Every reference nodule in each case goes through matching process. Unmatched predicted nodules result in a corresponding increase in the number of *FPs*.

To determine whether the predicted mark matches the reference standard (53) [this process is also called “mark-labeling” (54)], the flowchart of matching process is shown in Figure 2, and the specific details of mark-labeling methods are described as follows, and for a more intuitive schematic diagram, see Supplementary Figure A.1.

#### Center hit

In this study, the center point of the B-Box of the predicted nodule falls within the range of the corresponding reference nodule region as a successful detection match. If the predicted nodule has multiple slices, as long as one slice satisfies the above situation, the reference nodule can be recorded as successful detection. The successfully detected reference nodules and predicted nodules are recorded as true positive (*TP*) nodules. After all reference nodules participated in the mark matching process, the reference nodules and predicted nodules that are not successfully matched are recorded as false-negative (*FN*) and false-positive (*FP*) nodules, respectively. When a reference nodule meets the conditions for successful matching with multiple predicted nodules at the same time, the distance between the predicted nodule and the largest slice of the reference nodule is further compared. The predicted nodule with the smallest distance is recorded as the *TP* nodule, and other predicted nodules need to participate in the matching process of the remaining reference nodules. If a predicted nodule can be matched with multiple reference nodules, the first reference nodule is selected to match the predicted nodule according to the matching order. With respect to the predicted nodule with multiple slices, the definition of *TP, FN, FP* nodules, and the matching of multiple reference nodules with a predicted nodule, are handled in the same way in the three mark-labeling methods.

#### Center distance

For center distance scheme, the distance between the center point of the predicted nodule B-Box and that of the corresponding reference nodule B-Box is compared with a threshold. Such a threshold is varied which is adaptively set to the average radius of each reference nodule under matching, which is the maximum of one quarter of the sum of the long and short diameters across slices. If the distance is less than threshold, reference nodule is considered successfully detected. When a reference nodule meets the conditions for successful matching with multiple predicted nodules at the same time, their distance is further compared, and the predicted nodule with the shortest center distance is recorded as the *TP* nodule, and the remaining predicted nodules participate in the matching process of other reference nodules.

#### Area overlap

For “area overlap” rule, it is stipulated that the proportion of the overlapping part of the predicted nodule B-Box area and the reference nodule B-Box area to the reference nodule B-Box area is greater than a threshold as a successful detection, and the threshold is set to 0.5 empirically. When looking for a matching predicted nodule for a certain reference nodule, if there are multiple predicted nodules that can satisfy the aforementioned description of successful matching, their proportions are further compared, and the predicted nodule with the highest proportion is considered to match the reference nodule.

### Performance evaluation metrics

Recall, precision, and F_1_ score were selected to evaluate algorithm performance. The recall reflects the proportion of the correct nodules detected by the algorithm to the reference nodules, that is, whether the algorithm can find out as many reference nodules as possible. The accuracy reflects the proportion of the correct nodules detected by the algorithm to the nodules predicted by the algorithm itself, that is, whether the algorithm can predict the reference nodules as accurately as possible. The F_1_ score reflects an overall performance. For the definitions of *TP, FP* and *FN*, see the description in section Mark-labeling Methods. It can be seen that different mark-labeling methods may affect the judgement and quantities of *TP, FP*, and *FN*, thereby influencing the recall rate, precision, and F_1_ score values.

## Results

### Comparison between three mark-labeling methods

**Figure 3:**
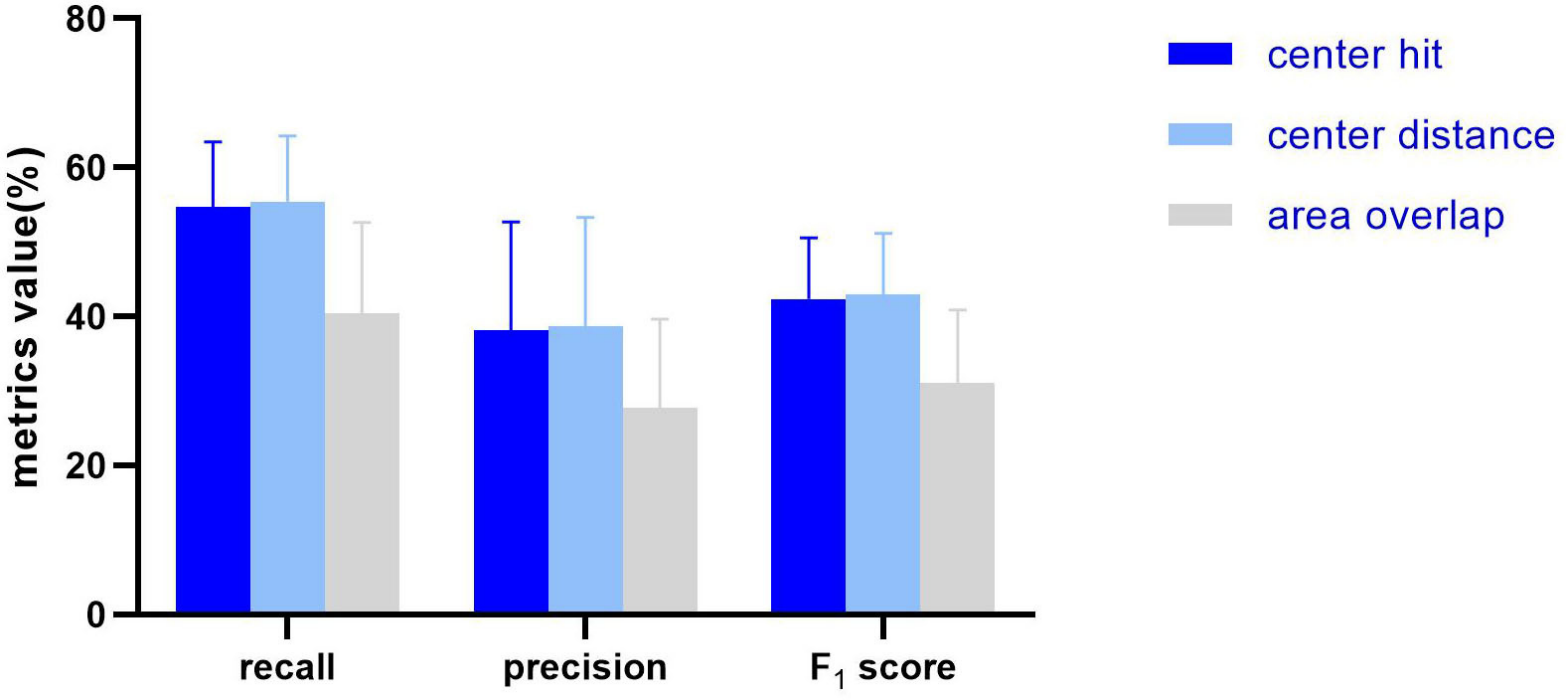
The histogram showing the average and standard deviation of the recall, precision, F_1_ score of the 10 AUTs according to three mark-labeling methods.

Test the detection performance of ten algorithms according to the three matching methods specified in chapter Mark-labeling Methods, and comprehend the impact of different testing methods on the metrics. The overall performance of the ten algorithms is represented by the mean ± standard deviation (%) of the metric values (Figure 3).

In general, the algorithm performance under center hit and center distance is higher than the area overlap, and the test results of center hit and center distance are very close (the mean of recall, precision, and F_1_ Score differ by 0.75, 0.5, and 0.57%, respectively). Compared with the center hit, the average value of the three indicators all drop by more than 10%. Among them, the recall dropped the most by 14.33%, the precision dropped by 10.44%, and the F_1_ score dropped by 11.26%.

**Figure 4:**
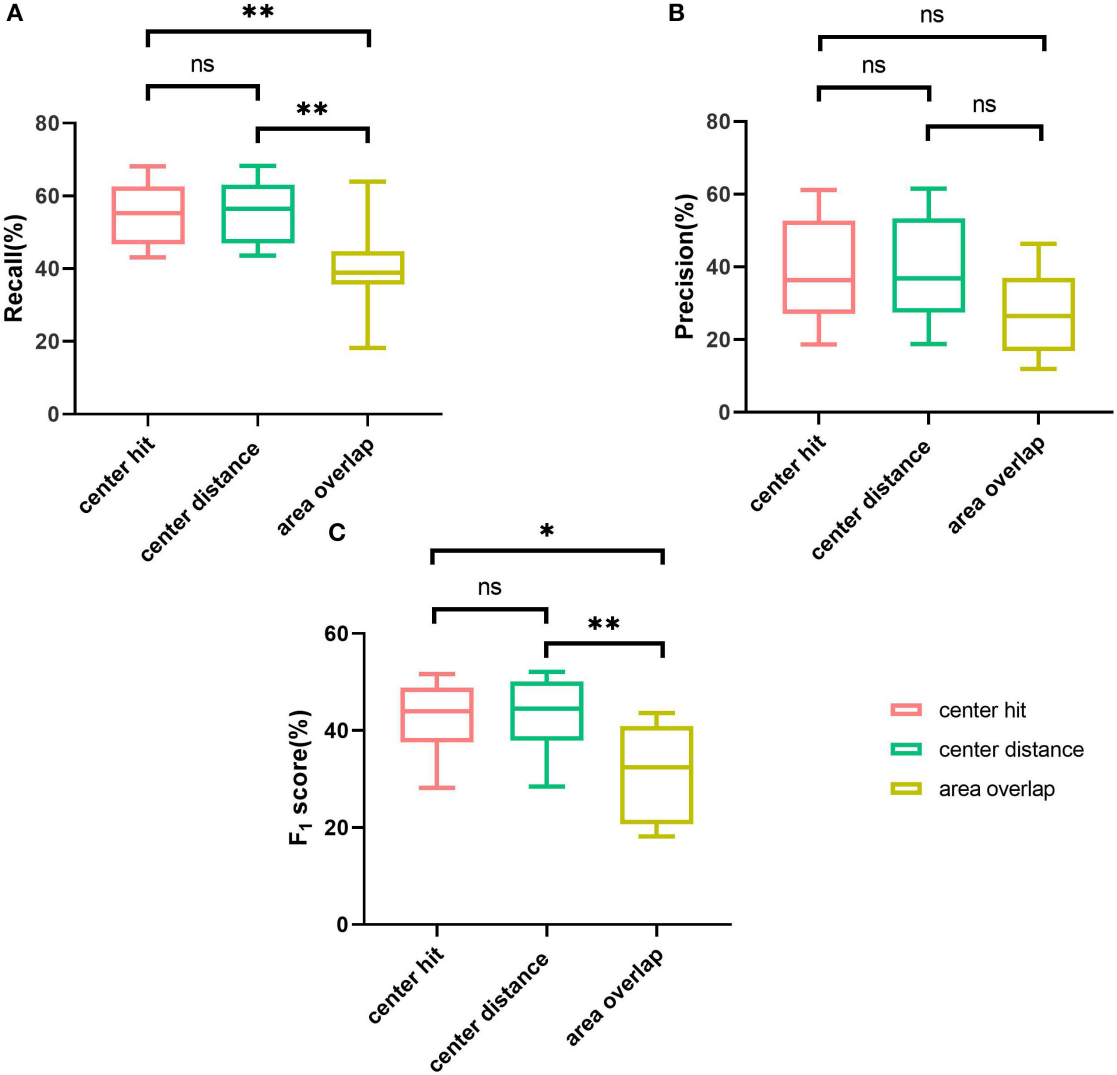
Comparison of three mark-labeling methods by boxplot. **(A)** The boxplot showing the range of the recall of the AUTs according to three mark-labeling methods. **(B)** The boxplot showing the range of the precision of the AUTs according to three mark-labeling methods. **(C)** The boxplot showing the range of the F1 score of the AUTs according to three mark-labeling methods. Note: **means *P* value is no more than 0.01, *means *P* value is no more than 0.05, ns means P value is greater than 0.05.

Further, different matching methods are regarded as different groups, and then the results under different matching methods are analyzed by the analysis of variance (ANOVA) and *t*-test. When *P* > 0.05, it is considered that there is no significant difference between groups. For recall (Figure 4A), significant differences are seen among the three groups (*P* = 0.0033), and through *t*-test analysis, there are significant differences between center hit and area overlap (*P* = 0.0075), and between center distance and area overlap (*P* = 0.0054). For center hit and center distance, there is no significant difference between two groups (*P* = 0.8503). Regarding the precision (Figure 4B), although the mean precision for the area overlap is lower than the other two matching methods, from the analysis of variance, there is no significant difference in the mean precision under the three matching methods (*P* = 0.152). Also, there is no significant difference between any two groups under the *t*-test (*P* = 0.9394 for center hit and center distance, *P* = 0.0954 for center hit and area overlap, *P* = 0.0829 for center distance and area overlap). As for F_1_ score (Figure 4C), the same as the variance analysis of recall, significant differences are seen among the three groups (*P* = 0.0080). And similarly, *t*-test results for any two groups show statistically significant differences between area overlap and center hit (*P* = 0.0120) and between area overlap and center distance (*P* = 0.0090). In the meanwhile, *P*-value between center hit and center distance is 0.8782 which means there is no significant difference between two groups.

**Figure 5:**
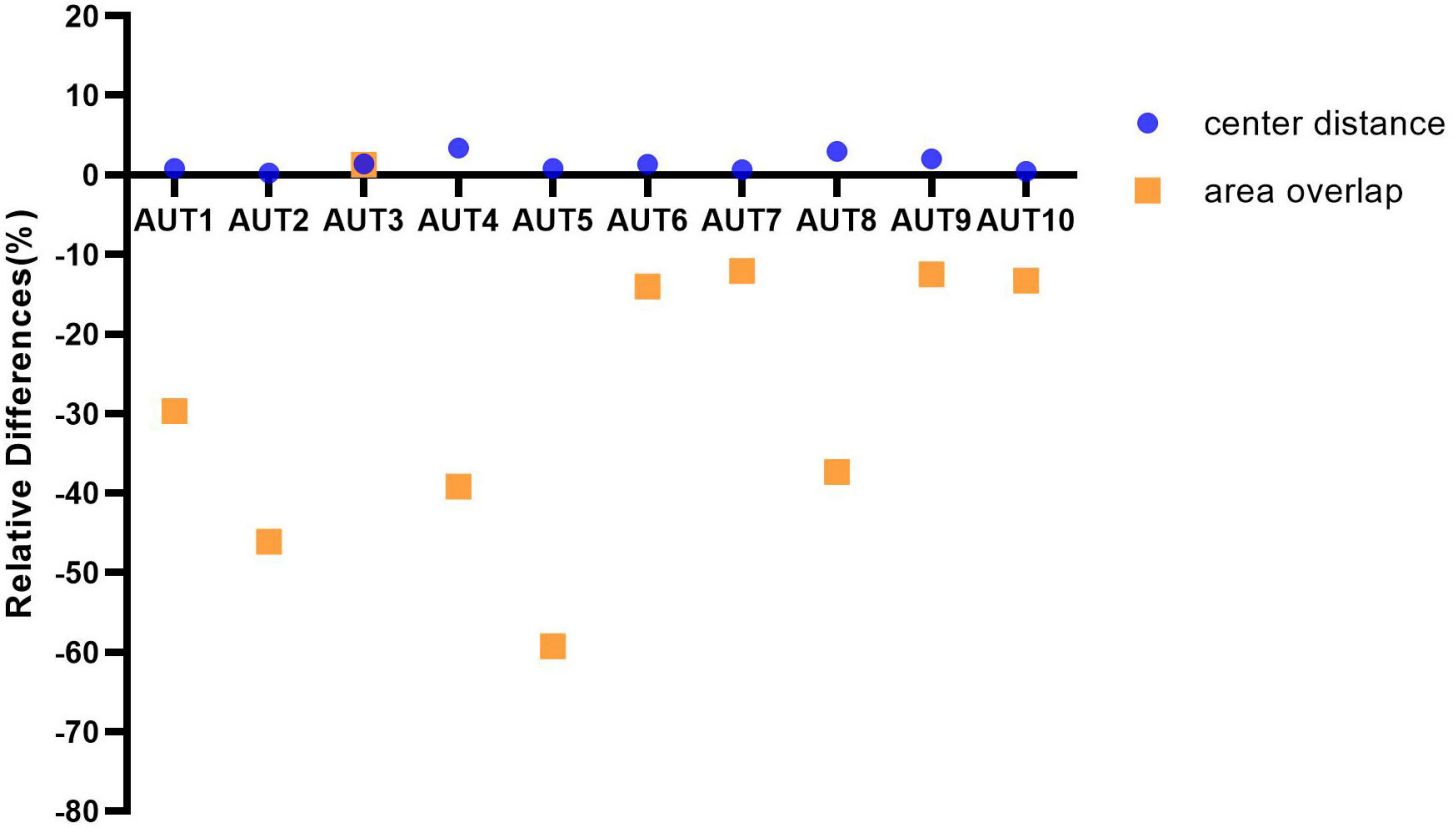
The graph depicting ten AUTs' relative differences of center distance and area overlap with respect to center hit, respectively.

In order to have a more intuitive impression of the specific performance differences of different AUTs under the three matching methods, the relative differences of ten AUTs under the three matching rules are further explored from the perspective of *TP* nodules. Specifically, test results under the “center hit” rule are chosen as the baseline. The relative changes for the other two matching rules are calculated, respectively (Figure 5). For example, the calculation formula of the relative difference (RD) of the recall under “center distance” rule is as follows.


(1) $$\begin{array}{l} \begin{array}{c} RD_{dist}=\frac{TP_{dist}-TP_{hit}}{TP_{hit}} \end{array} \end{array}$$

In Figure 5, the ten AUTs show varying degrees of difference for the three matching methods. The performance of the 90% (9/10) AUTs under “area overlap” rule is relatively lower than the other two matching methods. There is one AUT (AUT 5) that is particularly unaccustomed to the test method of area overlap, with a relative drop of as high as 59%. And it is worth mentioning that there is also an AUT (AUT 3) that maintains a high degree of consistency under three mark-labeling rules, and its performance under “area overlap” rule is slightly higher than that under “center hit” rule, which is fundamentally different from other AUTs. In addition, the performance of all AUTs under “center distance” rule is slightly higher than that under “center hit” rule, and the relative increase is as high as 3.34%. All AUTs show relatively optimal test results under “center distance” rule.

### Analysis of FN nodules

After comparing the differences in the evaluation metrics of AUTs under three mark-labeling rules objectively, in order to assess the quality of AUTs and try to find out the reasons for the general performance of the AUTs using this sequestered test set, further research is carried out to count the erroneous results of ten AUTs under “center hit” rule. The type and size of nodules may affect the performance test results, which were taken into account in experimental design. Focus on the reference nodules that were not successfully detected, namely *FN* nodules, features of *FN* nodules in the two dimensions of type and size were observed, and the common quality characteristics of AUTs were investigated. In addition, we selected the images of FN and TP nodules of four types of nodules with stronger medical significance to indicate the nodules that are difficult to detect and relatively easy to detect by the algorithms, see Supplementary Figures A.2–A.5.

#### Six types of nodules

**Figure 6:**
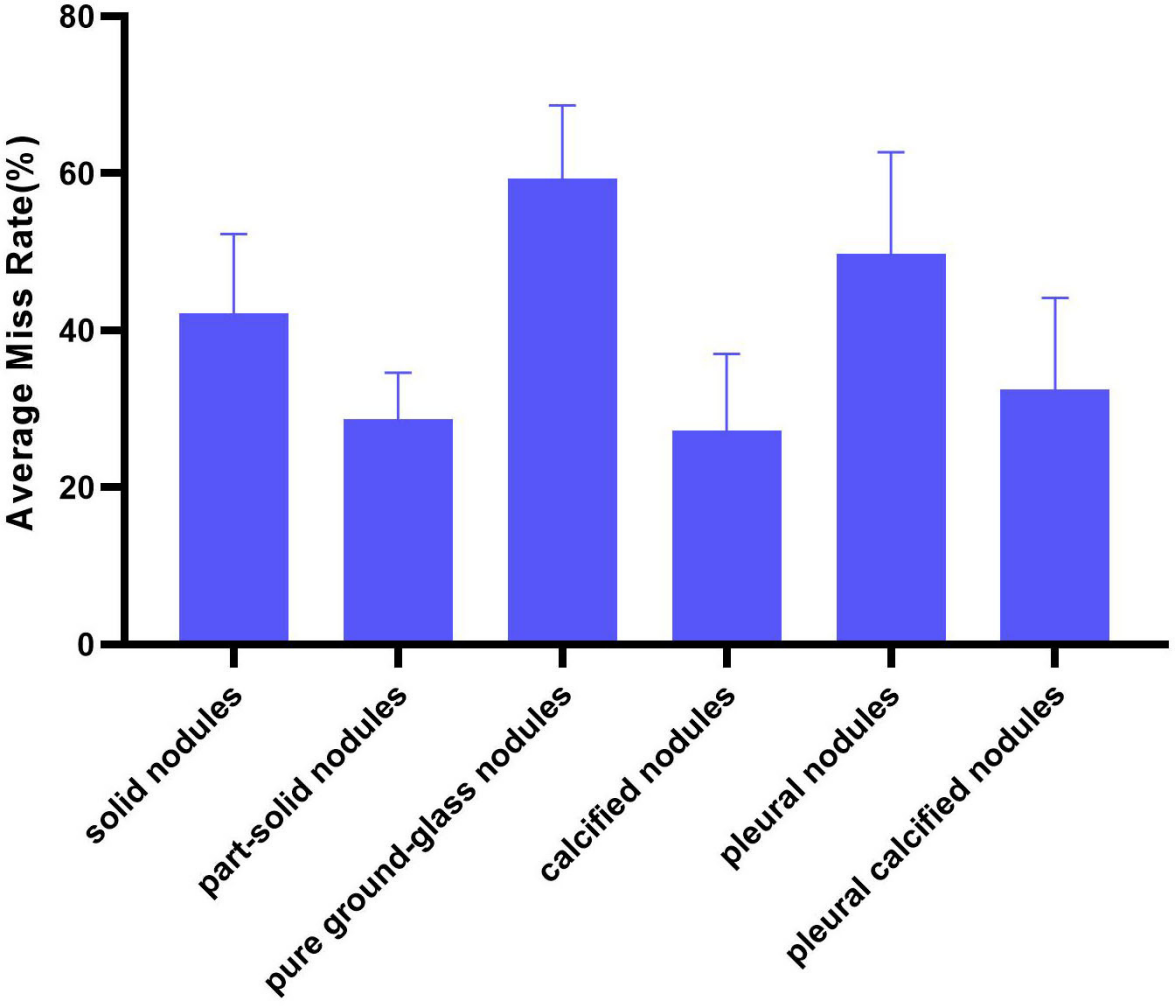
The average miss rate of AUTs on the six types of nodules under the center hit.

Divide the number of each type of *FN* nodules by the number of this type of reference nodules as the miss rate of AUT on this type of nodules. Then, the miss rate of all AUTs for a certain type of nodules is expressed in the form of mean ± standard deviation (%) to illustrate the overall situation of AUTs' miss detection of this type of nodules (Figure 6). And overall miss situation of different nodule types can be compared.

In terms of nodule types, AUTs can detect part-solid nodules and calcified nodules more accurately than other types, with an average miss rate of 28.64 and 27.28%, respectively. Especially for part-solid nodules, the fluctuation of detection performance of different AUTs on such nodules is also the smallest (standard deviation is 5.96%). For these AUTs, the most challenging nodule type is pure ground-glass nodule, with an average miss detection rate of over 50% (as high as 59.32%, twice as many as solid nodules), followed by pleural nodules and solid nodules, with an average miss rate of 49.80 and 42.21%.

#### Combine size and type

**Figure 7:**
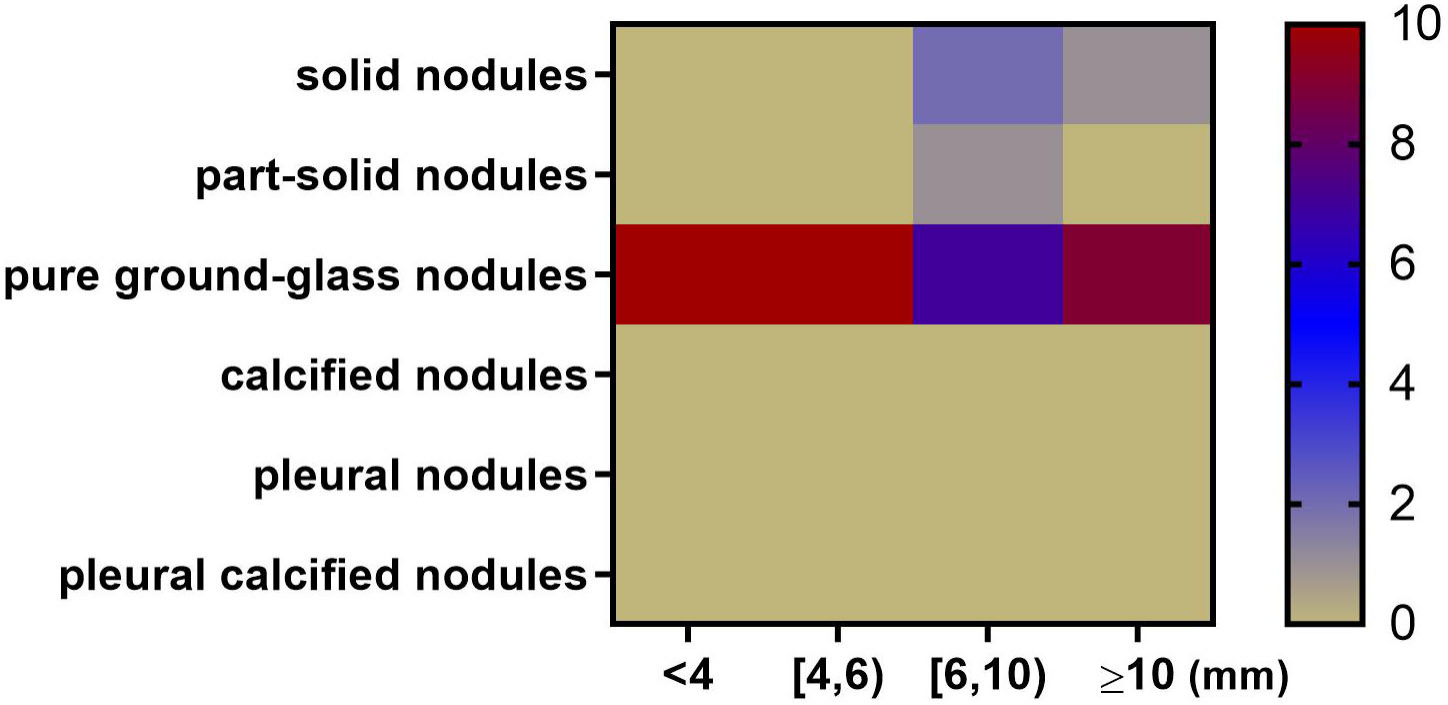
Heatmap depicting nodule types and diameters most likely to be missed by 10 algorithms. The x axis represents nodule size range. The y axis represents nodule type. For example, when the nodule diameter is between 6 and 10 mm, 7 AUTs are most likely to miss pure ground-glass nodules; 2 AUTs are most likely to miss part-solid nodules; 1 AUT is most likely to miss solid nodules.

The dimension of size was added to further refine the types that are likely to be missed in different size ranges. The specific method is to plot the miss rate of each type of nodules against the range of average diameter, including 4 bins [ < 4, (4, 6), (6, 10), ≥10 mm] for each AUT, respectively. Within each bin, nodule types with highest miss rate for each AUT are counted and shown as a heatmap (Figure 7).

In general, among all nodule diameter ranges, pure ground-glass nodules are the most likely to be missed. For nodules < 6 mm in diameter, all AUTs have the highest miss detection rate for pure ground-glass nodules among all types. In the diameter range from 6 to 10 mm, pure ground-glass nodules are the most difficult type to be detected for 7 AUTs. Part-solid nodules are the most difficult type to be detected for one AUT. Solid nodules in this size range are most difficult for two AUTs. For large nodules larger than 10 mm in size, 90% (9/10) of AUTs need to continue to make efforts to detect pure ground-glass nodules, there is also an AUT that needs to detect large solid nodules more accurately.

## Discussion

With the development of AI technology, more computer-aided detection products for pulmonary nodules may enter the market in the future. While verification and validation activities are mainly conducted by manufacturers, it is important for regulators and public stakeholders to understand the algorithm performance in a more comparable manner. From the perspective of third-party testing, it may be helpful to explore a pathway to build test set and compare products directly, objectively and quantitatively.

This study demonstrated a centralized method to build test set and conduct algorithm performance testing. Data was randomly sampled from diverse hospitals and regions according to the design input. Annotators were recruited publicly through qualification exams and randomly grouped to conduct annotation. The whole process relies on the same standardized procedure. The workflow is different from conventional multicenter study and may decrease variation of annotation among different hospitals.

Using the same test set, different AUTs are tested and compared quantitatively. The testing results indicated that specific mark-labeling method would affect interpretation of algorithm performance. This study shows that, among the three mark-labeling methods adopted in the experiments, ten AUTs under the method of center distance showed the highest precision and recall, as performance metrics of computer-aided detection. The “center hit” method showed intermediate results. The “area overlap” method showed the worst results. From the perspective of clinical application, the mark-labeling methods are associated with follow-up operations after image analysis. For example, if robotically assisted surgery needs information from computer-aided detection of pulmonary nodules, it would be necessary for the AUT to export the position of predicted nodule, so the “center distance” approach is favorable. Under the context of radiotherapy, the “area overlap” approach is preferred. It may be helpful to choose mark-labeling rule according to the intended use and usage scenarios of the product.

In this study, it seems that the average recall of the ten AUTs is lower than results reported in other literature (25–27). There are several underlying reasons. First, the test set used in this study is independent and isolated from the training or tuning process of AUTs. In other literature, AUTs may be trained and tuned on a subset of a large data set and then tested on another subset. The correlation between training and testing data may facilitate the model to achieve better testing results. Therefore, the test set in this paper seems more challenging and helps reflect the generalizability of algorithm. Second, the annotation of the test set is based on a centralized and relatively strict procedure, which requires intense support from experienced radiologists. For developers of the AUTs, however, their training sets and tuning sets are prepared spontaneously. Annotation activities and results may have difference.

For the evaluation metrics, the FROC curve was not adopted (other researchers may have chosen) because each developer has fixed the optimal detection threshold before providing the algorithm. In order to be more in line with the actual clinical use scenarios of the product, evaluation metrics such as recall on its specified detection threshold were calculated.

Based on the above results, the algorithm errors were further compared among different AUTs and analyzed the trend. False-negative nodules are chosen as the target, and we observed what type and size of nodules are more likely to be missed by AUTs, resulting in a lower recall by the algorithm. Based on the research results, manufacturers are encouraged to pay attention to the accuracy in the detection of solid nodules, pure ground-glass nodules and pleural nodules in the development stage. Especially for pure ground-glass nodules of various sizes, the low detection accuracy of such nodules is the main reason for the poor performance of AUTs. It is necessary to consider taking related technical methods (such as increasing its proportion in the training set, using better tuned algorithms, etc.) to improve the detection performance of the product on these three types of nodules and small nodules. At the same time, how to reduce the number of false positive nodules also needs to be considered in the development process, and there is also a trade-off between recall and precision.

In summary, this paper systematically described a standardized workflow to build test sets and conduct algorithm testing in a third-party manner. The test set construction covered data collection, data curation, annotation, annotator management, which followed data quality management standards (49). If organizations follow this workflow, the comparability of data sets may be improved, since each step is well defined and the rule is relatively transparent. The algorithm testing section compared both performance metrics and trend of algorithm errors among different products, which also provided useful evidence to enrich the perspective of product evaluation and comparison.

There are several limitations in this study. First, while three mark-labeling rules are compared, it is difficult to assign the specific threshold of “center distance” rule or “area overlap” rule. The thresholds used in the experiments are selected empirically. It may not represent the requirement from clinical users' perspective. More discussion on the threshold selection should be made in the future. Consensus is needed to propose clear requirement on how computer-aided detection products should present the algorithm output. Second, this study compared different algorithm outputs on a sequestered test set in a black box manner, providing barely no clue to evaluate the process of algorithm design. It is difficult to further discuss the advantages and disadvantages of model design according to such test results, since there may be implicit discrepancies in the training sets, parameter settings, and training methods among developers in the research and development stage. Third, efficiency is not compared among different AUTs since they operated on separate servers that were provided by developers. Since medical device manufacturers would claim their own requirement on computation resource during premarket application, it may be helpful to define a benchmark to further evaluate algorithm efficiency based on consensus of manufacturers and clinical users.

In the future, more work will be conducted to evaluate the quality and comparability of test sets, which may further support standardization of testing methods and provide technical reference for the regulation of such products.

## Data availability statement

The original contributions presented in the study are included in the article/Supplementary material, further inquiries can be directed to the corresponding authors.

## Author contributions

HW and NT performed algorithm testing experiments and wrote the manuscript draft. CZ and YH analyzed the data. XM and JL contributed to the data collection. All authors read, contributed to the research design, and approved the final manuscript.
