# Supplementary material for: Practice toward standardized performance testing of computer-aided detection algorithms for pulmonary nodule
Source: Front Public Health. 2022 Dec 7;10:1071673. doi: 10.3389/fpubh.2022.1071673 (PMC9768365; doi:10.3389/fpubh.2022.1071673)
Supplement: Supplementary file 1 [file Data_Sheet_1.pdf]

Supplementary material

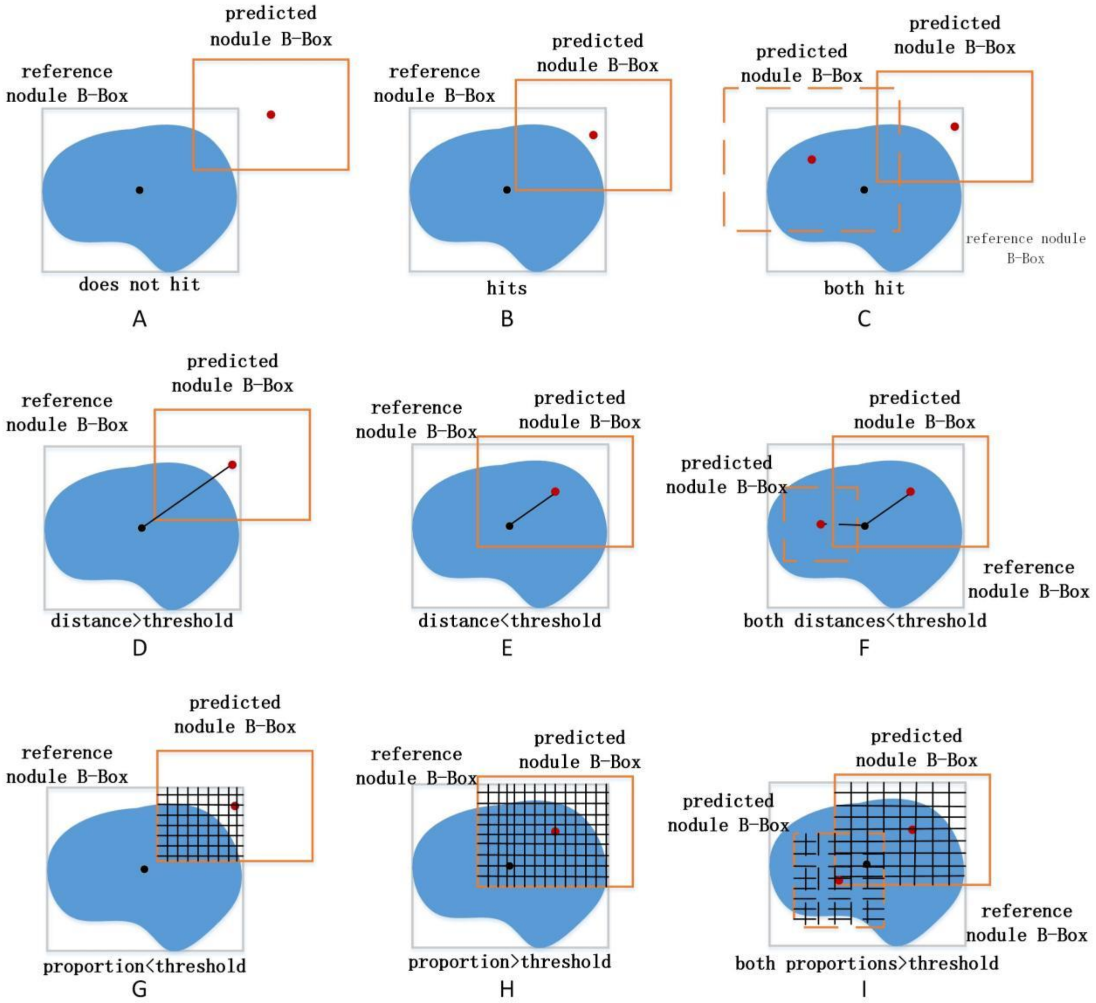

Figure A.1 Schematic diagram depicting the three situations of unsuccessful match, successful match, and multiple matches according to three mark-labeling methods (take a slice of the nodule as an example). The gray box and blue region represent the B-Box and region of the reference nodule; the predicted nodule is represented by the orange box (solid or dashed lines); the black dot represents the center of the reference nodule B-box, and the red dot represents the center of the predicted nodule B-box. A single black line represents the distance between the two centers. The area of black grid lines indicates the overlapping area of the predicted and reference nodules. The graph in the first row (A-C) shows three situations according to "center hit". The graph in the second row (D-F) shows three situations according to "center distance". The graph in the third row (G-I) shows three situations according to "area overlap". The graph in the first column(A, D, G) is the situation where the predicted nodule failed to match the reference nodule. The graph in the second column(B, E, H) is the situation where the predicted nodule successfully matches the reference nodule. The graph(C, F, I) in the third column is the situation where more than one predicted nodules can match the reference nodule, but only one predicted nodule can be recorded as TP (predicted nodules represented by red dashed box in C and F and red solid box in I) according to the corresponding rules.

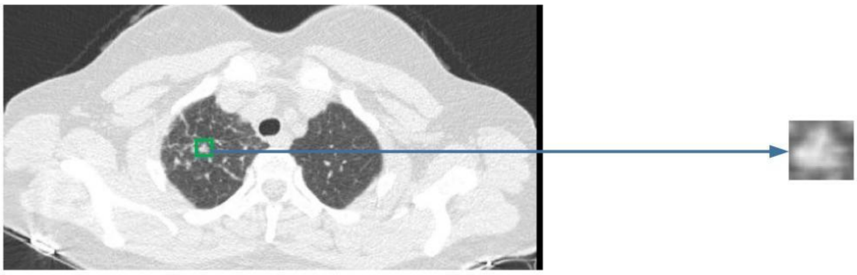

A

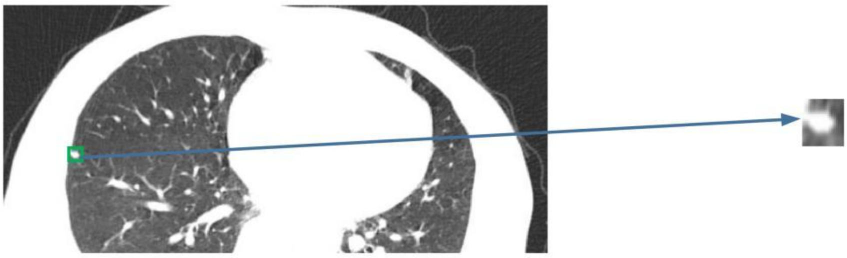

B

Figure A.2 Example of solid nodules that are particularly difficult to detect and relatively easy to detect. The left column is the image slice where the largest cross section of the nodule is located. The right column is a zoomed image of the largest cross section of the nodule. The solid nodule in A is a particularly difficult nodule to detect (no AUT succeeded). The solid nodule in B is relatively easy nodule to detect (all AUTs succeeded).

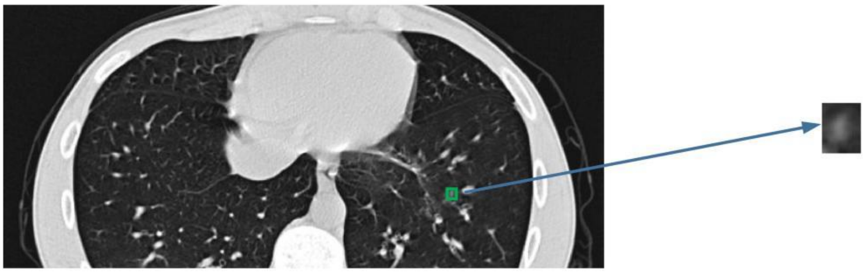

A

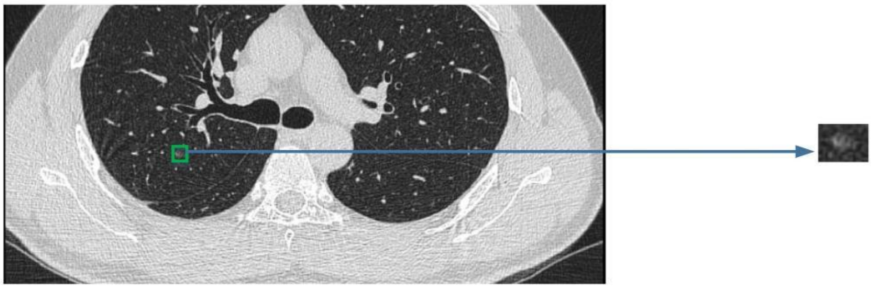

B

Figure A.3 Example of part-solid nodules that are particularly difficult to detect and relatively easy to detect. The left column is the image slice where the largest cross section of the nodule is located. The right column is a zoomed image of the largest cross section of the nodule. The part-solid nodule in A is a particularly difficult nodule to detect (no AUT succeeded). The part-solid nodule in B is relatively easy nodule to detect (all AUTs succeeded).

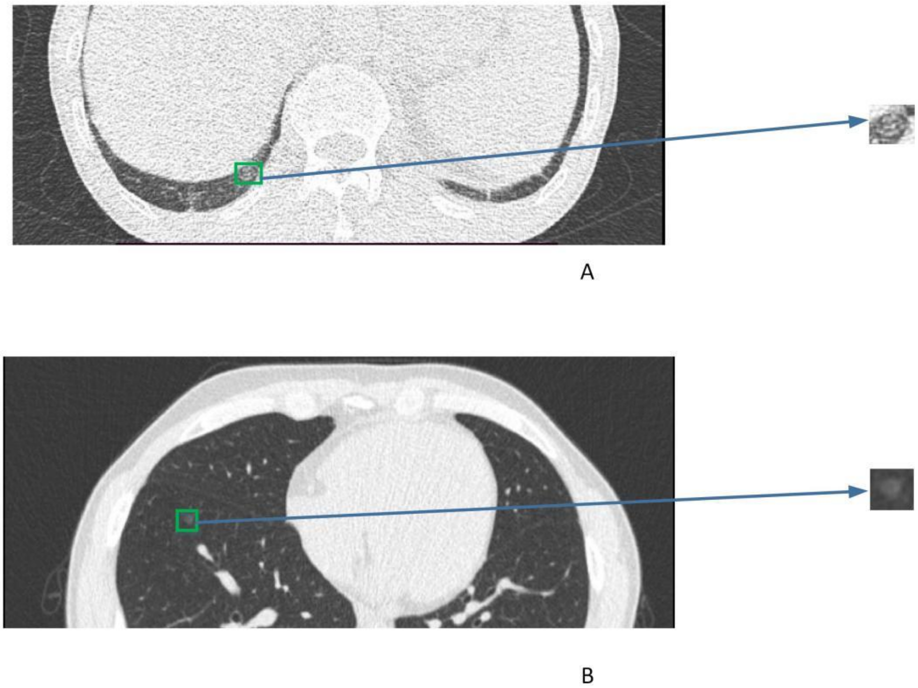

Figure A.4 Example of pure ground-glass nodules that are particularly difficult to detect and relatively easy to detect. The left column is the image slice where the largest cross section of the nodule is located. The right column is a zoomed image of the largest cross section of the nodule. The pure ground-glass nodule in A is a particularly difficult nodule to detect (no AUT succeeded). The pure ground-glass nodule in B is a relatively easy nodule to detect (all AUTs succeeded).

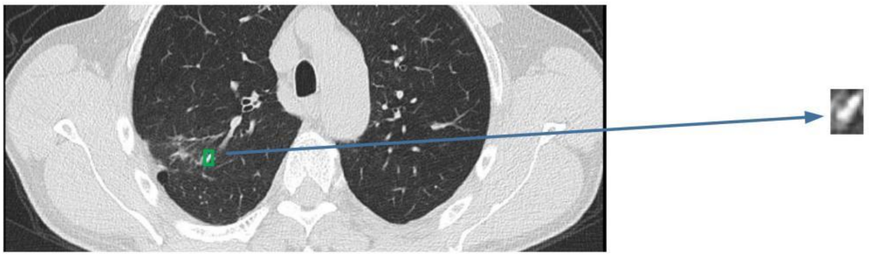

A

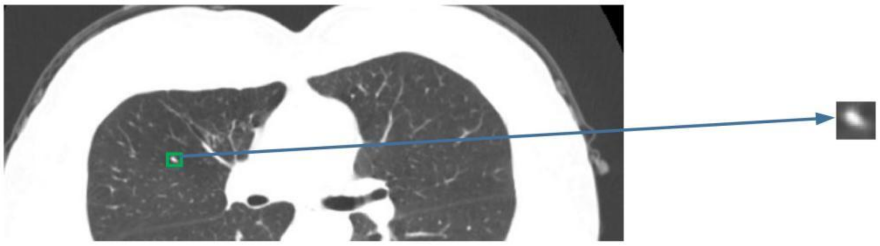

B

Figure A.5 Example of calcified nodules that are particularly difficult to detect and relatively easy to detect. The left column is the image slice where the largest cross section of the nodule is located. The right column is a zoomed image of the largest cross section of the nodule. The calcified nodule in A is a particularly difficult nodule to detect (no AUT succeeded). The calcified nodule in B is relatively easy nodule to detect (all AUTs succeeded).
